# Supplementary material for: 3D printable and biocompatible PEDOT:PSS-ionic liquid colloids with high conductivity for rapid on-demand fabrication of 3D bioelectronics
Source: Nat Commun. 2024 Jul 11;15:5839. doi: 10.1038/s41467-024-50264-6 (PMC11239939; doi:10.1038/s41467-024-50264-6)
Supplement: Supplementary file 1 — Supplementary Information [file 41467_2024_50264_MOESM1_ESM.pdf]

Supplementary Information

3D Printable and Biocompatible PEDOT:PSS-Ionic Liquid Colloids with High conductivity for Rapid on-demand Fabrication of 3D Bioelectronics

Byungkook Oh[1,†], Seunghyeok Baek[1,†], Kum Seok Nam[2,†], Changhoon Sung[2], Congqi Yang[2], Young-Soo Lim[3], Min Sang Ju[4], Soomin Kim[1], Taek-Soo Kim[4], Sung-Min Park[3,5,6,7], Seongjun Park[1, 2, 8]*, Steve Park[1,8]*

†: These authors contributed equally: Byungkook Oh, Seunghyeok Baek and Kum Seok Nam

*: Corresponding Author

Corresponding Author e-mail : spark19@kaist.ac.kr (Seongjun. P.); stevepark@kaist.ac.kr (Steve.P.)

1. Department of Materials Science and Engineering, Korea Advanced Institute of Science and Technology (KAIST), 291 Daehak-ro, Yuseong-gu, Daejeon, Republic of Korea
2. Department of Bio and Brain Engineering, Korea Advanced Institute of Science and Technology (KAIST), 291 Daehak-ro, Yuseong-gu, Daejeon, Republic of Korea
3. Department of Convergence IT Engineering (CiTE), Pohang University of Science and Technology (POSTECH), 77 Cheongam-ro, Nam-gu, Pohang-si, Gyeongsangbuk-do, Republic of Korea
4. Department of Mechanical Engineering, Korea Advanced Institute of Science and Technology (KAIST), 291 Daehak-ro, Yuseong-gu, Daejeon, Republic of Korea
5. Department of Electrical Engineering, Pohang University of Science and Technology (POSTECH), 77 Cheongam-ro, Nam-gu, Pohang-si, Gyeongsangbuk-do, Republic of Korea
6. Department of Mechanical Engineering, Pohang University of Science and Technology (POSTECH), 77 Cheongam-ro, Nam-gu, Pohang-si, Gyeongsangbuk-do, Republic of Korea
7. Institute of Convergence Science, Yonsei University, Seoul, Republic of Korea
8. KAIST Institute for NanoCentury, 291 Daehak-ro, Yuseong-gu, Daejeon, Republic of Korea

Results

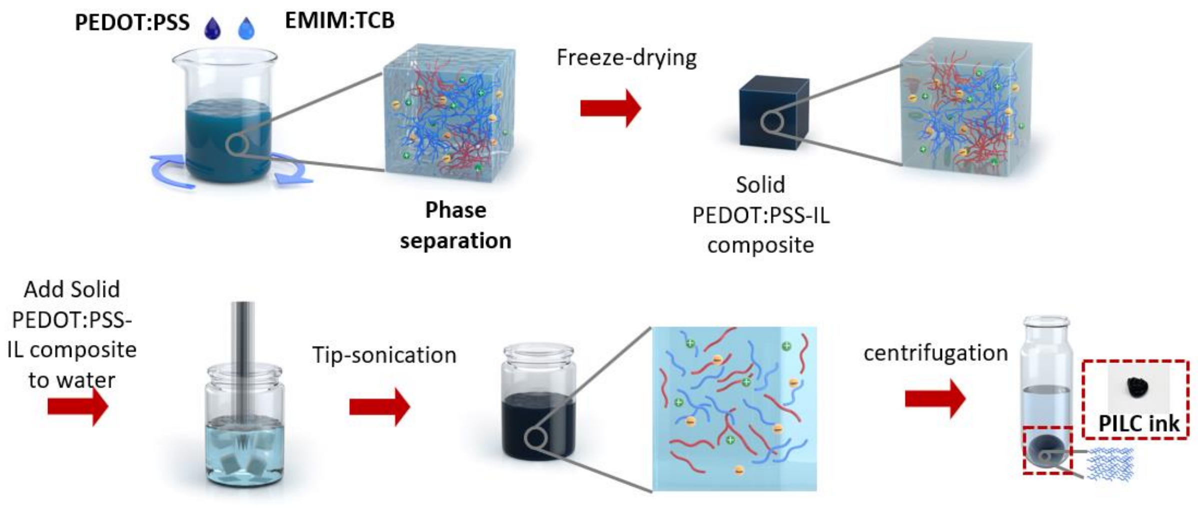

Supplementary Fig. 1 | Fabrication methodology of the 3D printable, biocompatible and highly conductive PEDOT:PSS-ionic liquid colloidal (PILC) inks. A one-shot strategy for general purpose PEDOT:PSS ink in versatile applications.

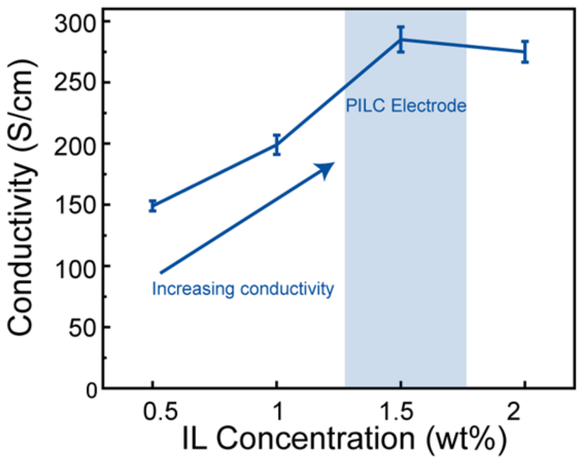

Supplementary Fig. 2 | Optimized conductivity with various ionic liquid (IL) concentrations. The conductivity of the PILC ink increased with increasing IL concentration. Data represents as mean values ± SD (n = 4 independent experiments).

Ref	Viscosity (Pa*s)	Yield stress (Pa)	Storage modulus (Pa)	Conductivity (S/cm)	Intrinsic biocompatability	3D-Printed Layers (aspect ratio)	Water content (%)	Printing resolution (um)
[1]	$5*10^2$	10^2	$5*10^3$	28 (dry) / 155 (wet)	X (>24 hr post annealing)	20 (N/R)	87	30
[2]	23	N/R	N/R	0.022	N/A (> 1.5 hr post-treatment)	N/A	260	94
[3]	10^2	10^4	$5*10^3$	858.1 (Post-acid treatment)	N/A	25 (N/R)	N/R	50
[4]	$3*10^3$	$4*10^4$	$4*10^3$	72 (wet) / 654 (Post-acid treatment)	N/A	N/R (8~15)	N/R	15
[5]	100	N/R	N/R	11	N/A (> 24 hr post-treatment)	N/A	80	100
[6]	15	N/R	10^3	0.0159	N/A (> 1 hr post-treatment)	N/A	N/R	150
[7]	10^3	10^3	330	1,200 (Post-acid treatment)	N/A	10 (N/R)	N/R	50
[8]	10^2	10^3	10^3	0.09	N/A (> 24 hr post-treatment)	N/A	75	80
**This Work**	10^6	10^5	10^5	286	O	>100 (>4)	28.75	50

N/A : Not Applicable
N/R : Not Reported

Supplementary Table. 1 | Comparison with previous reported papers using printable PEDOT:PSS inks

Ref	Method	Young's modulus (kPa)	Tensile strength (MPa)	Stretchability (%)	Charge Storage Capacity (mC cm^{-2})	Initial Conductivity (S/cm)
[1]	Nozzle printing	1,100	N/R	N/R	N/R	28 (dry)<br>155 (wet)
[2]	Liquid-in-Liquid Printing	41.2 - 3,875	1 - 2.5	76 - 800	N/R	0.022
[5]	Nozzle printing	1,000	0.5 - 3.5	400	6	11
[6]	Nozzle printing	5 - 65	110	349	12.37	0.0159
[8]	Nozzle printing	650	0.9	120	5.83	0.09
[9]	Photolithography	2,000	0.5 - 2.2	15 - 35	60	20-40
[10]	Molding	25	130	610	80	247
[11]	Laser-Induced hase separation	120	15	15	32.13	670
**This Work**	Nozzle printing	750	0.139	88.6	2D : 47.04<br>3D : 104.827	286

N/R : Not Reported

Supplementary Table. 2 | Comparison with previous reported papers using PEDOT:PSS inks

Ref	Method	Resolution (um)	2D *In / Ex-vivo Application*	3D Suspended Structure	3D *In-vivo Application*
[1]	Nozzle printing	30	O (*In-vivo*)	X	X
[2]	Liquid-in-Liquid Printing	94	X	O	X
[4]	Nozzle printing	15	X	O	X
[5]	Nozzle printing	100	O (*In-vivo*)	X	X
[6]	Nozzle printing	150	O (*Ex-vivo*)	X	X
[8]	Nozzle printing	80	O (*In-vivo*)	X	X
[10]	Molding	N/R	O (*In-vivo*)	X	X
[11]	Laser-Induced phase separation	6	O (*In-vivo*)	X	X
[12]	orthogonal photochemistry printing	100	O (*Ex-vivo*)	O	X
[13]	Coating	N/R	O (*In-vivo*)	X	X
[14]	Laser cutting	150	O (*In-vivo*)	O	X
**This Work**	Nozzle printing	50	O *(In-vivo & Ex-vivo)*	O	O

N/R : Not Reported

Supplementary Table. 3 | Comparison of versatility with previous papers using PEDOT:PSS inks

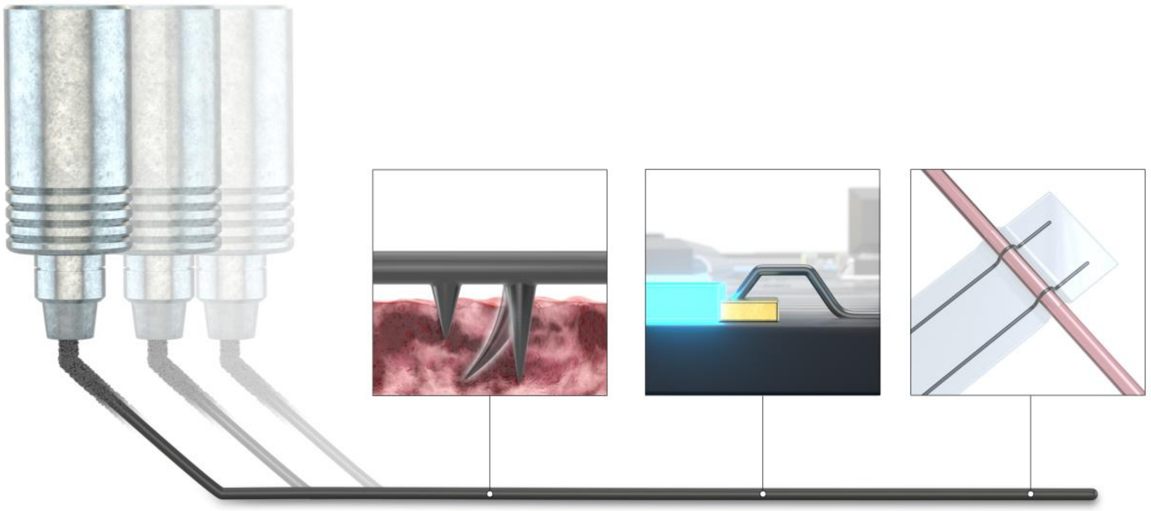

Supplementary Fig. 3 | Schematic illustration of versatile applicability of PILC inks from 2D *In-vivo / Ex-vivo* electrode and 3D diagonally printed circuit lines to 3D *In-vivo* electrodes. On-demand and versatile applicability of PILC inks through the rapid fabrication of implantable bioelectronics, skin bioelectronics and 3D circuit boards.

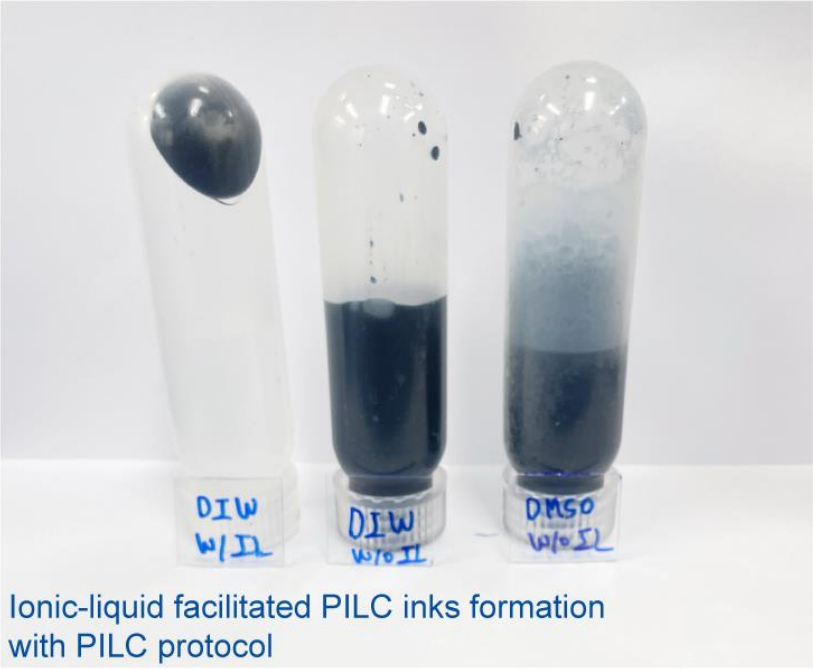

Supplementary Fig. 4 | Effect of ionic liquid on centrifugal phase separation. Photo of centrifugal phase separation of when freeze-dried PEDOT:PSS-ionic liquid composite used as solute (left), Photos of no phase separation when freeze-dried pristine PEDOT:PSS used as solute (middle) or freeze-dried water-insoluble pure PEDOT:PSS (freeze-dried PEDOT:PSS-DMSO composite) used as solute (right).

To confirm the effect of ionic liquid in PILC ink formation, all samples were prepared according to the PILC ink fabrication protocol with only the solute changed. Freeze-dried PEDOT:PSS-ionic liquid composite (left), freeze-dried PEDOT:PSS (middle) and freeze-dried PEDOT:PSS-DMSO composite (right) use as solute for each sample. After centrifugation, only the sample containing freeze-dried PEDOT:PSS-ionic liquid showed ink formation.

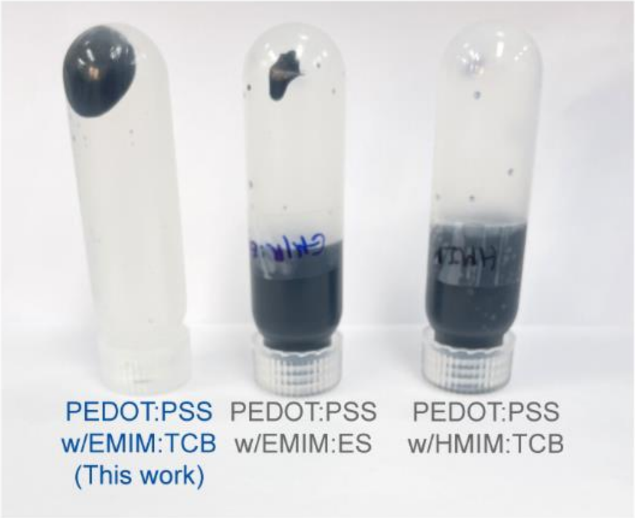

Supplementary Fig. 5 | Centrifuge effects of PEDOT:PSS and other ionic liquid (EMIM:ES and HMIM:TCB). Photo of centrifugal phase separation of when EMIM:TCB ionic liquid is used in order to make PILC ink (left), Photos of no phase separation when EMIM:ES (middle) and HMIM:TCB (right) ionic liquid are used in order to make PILC ink.

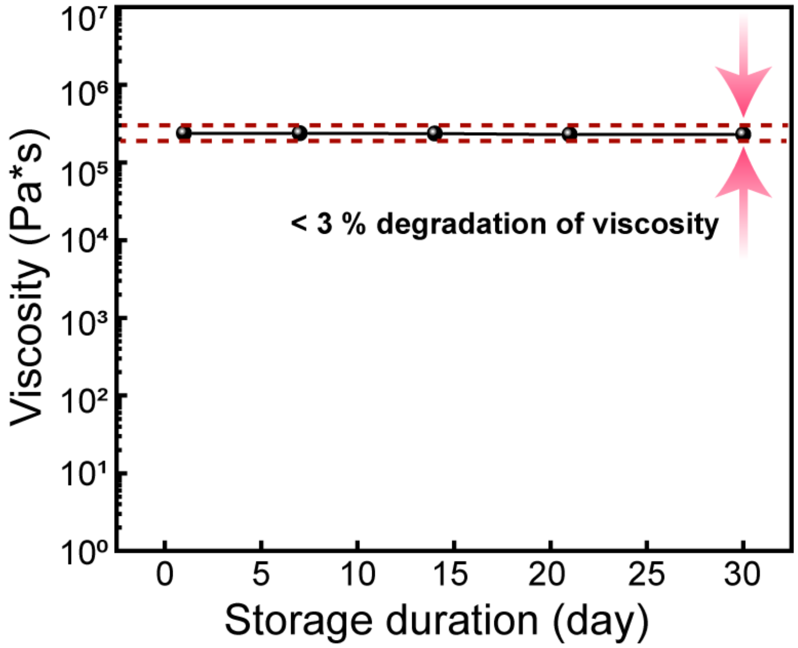

Supplementary Fig. 6 | Stable rheology of PILC inks. Viscosity of PILC inks over 30 days of storage at room temperature.

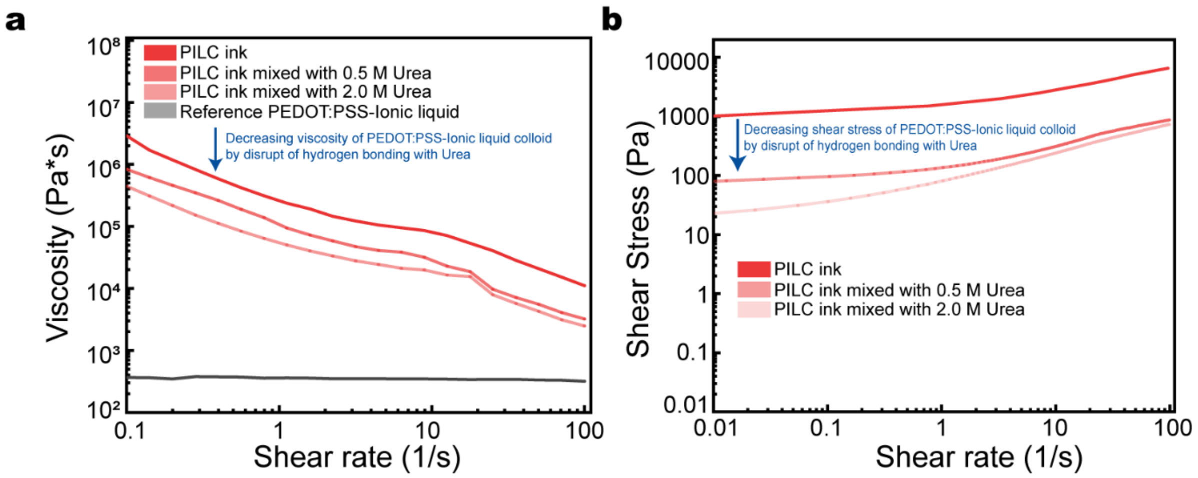

Supplementary Fig. 7 | Shear-thinning behavior of PILC inks at various urea concentrations (0.5 M and 2.0M). a, Viscosity versus shear rate with various concentrations of urea and PILC pellets. **b,** Shear stress versus shear rate with various concentrations of urea and PILC pellets.

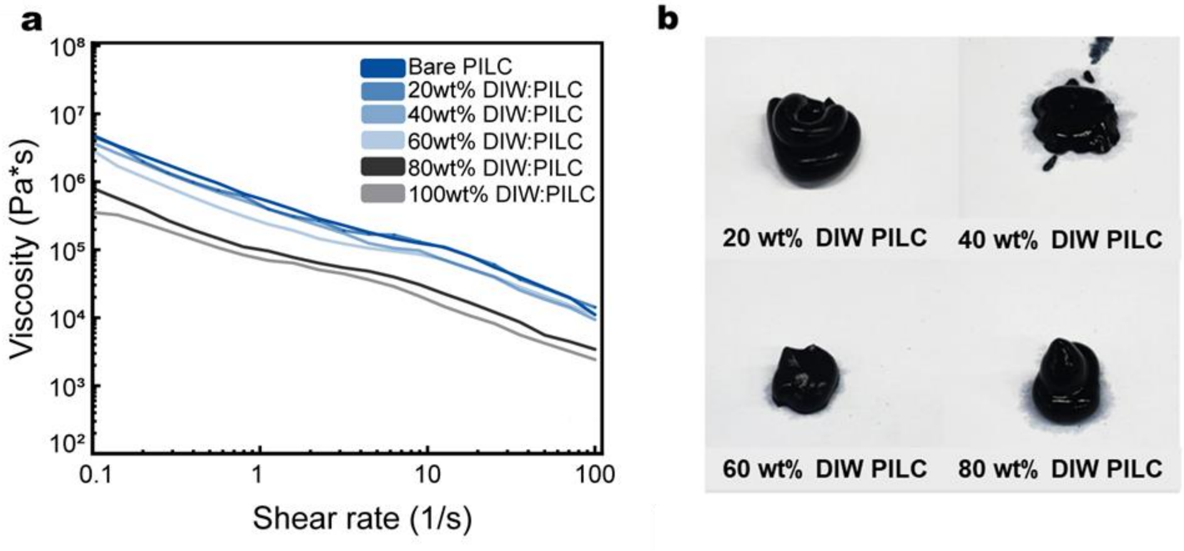

Supplementary Fig. 8 | Rheological properties of PILC inks at various concentrations. a, Viscosity versus shear rate with various concentrations of deionized water (DIW) to PILC pellet. The viscosity of PILC inks can be controlled with dispersion in water for various on-demand applications. **b,** Images of extruded PILC inks with various concentrations.

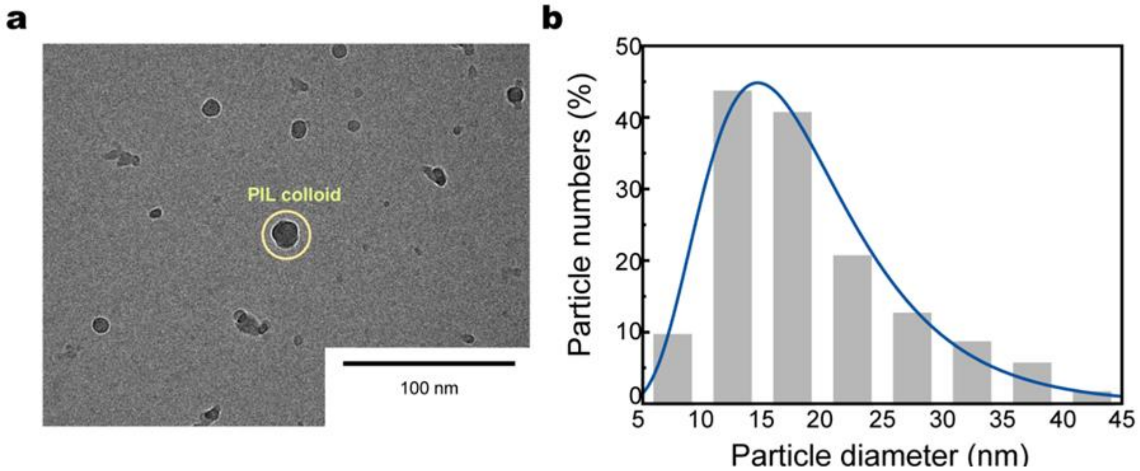

Supplementary Fig. 9 | Cryo-TEM image of PILC inks and their size distribution. a, Cryo-TEM image of PILC ink. **b,** PILC ink particle diameter distribution.

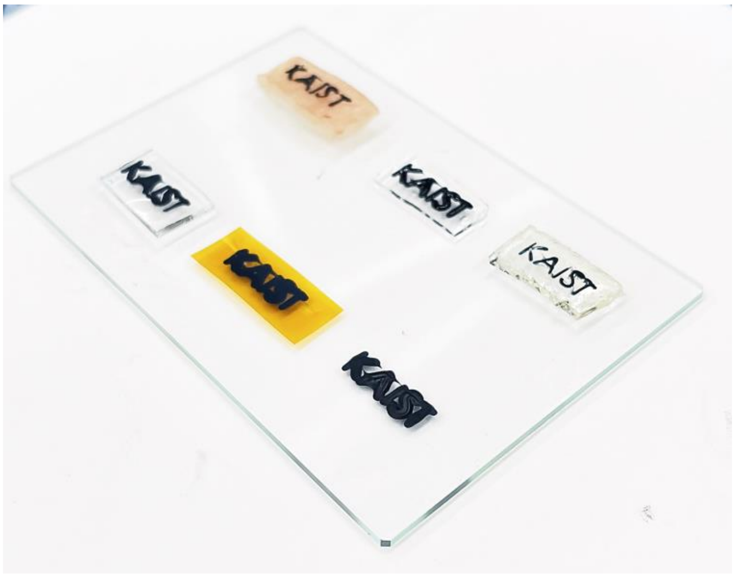

Supplementary Fig. 10 | Printing of PILC ink on various surfaces. Image of PILC inks printed on various films ranging from superhydrophobic film to hydrogels and porcine skin without any prior surface modification.

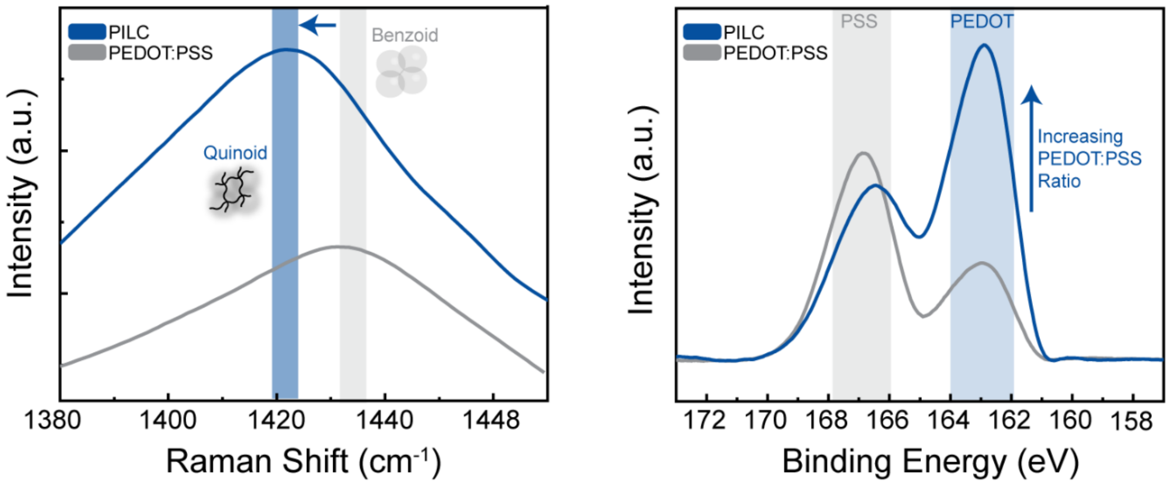

Supplementary Fig. 11 | PILC mechanism for high conductivity. Raman spectrum (left) and X-ray photoelectron spectroscopy (XPS) analysis (right) of PILC. Raman spectrum demonstrates a structural transition of PEDOT:PSS from benzoid to quionoid conformation due to ionic liquid-facilitated phase separation. XPS reflects an increase in the S *2p* peak of the PEDOT phase in a PILC ink at 162-167 eV compared to pristine PEDOT:PSS.

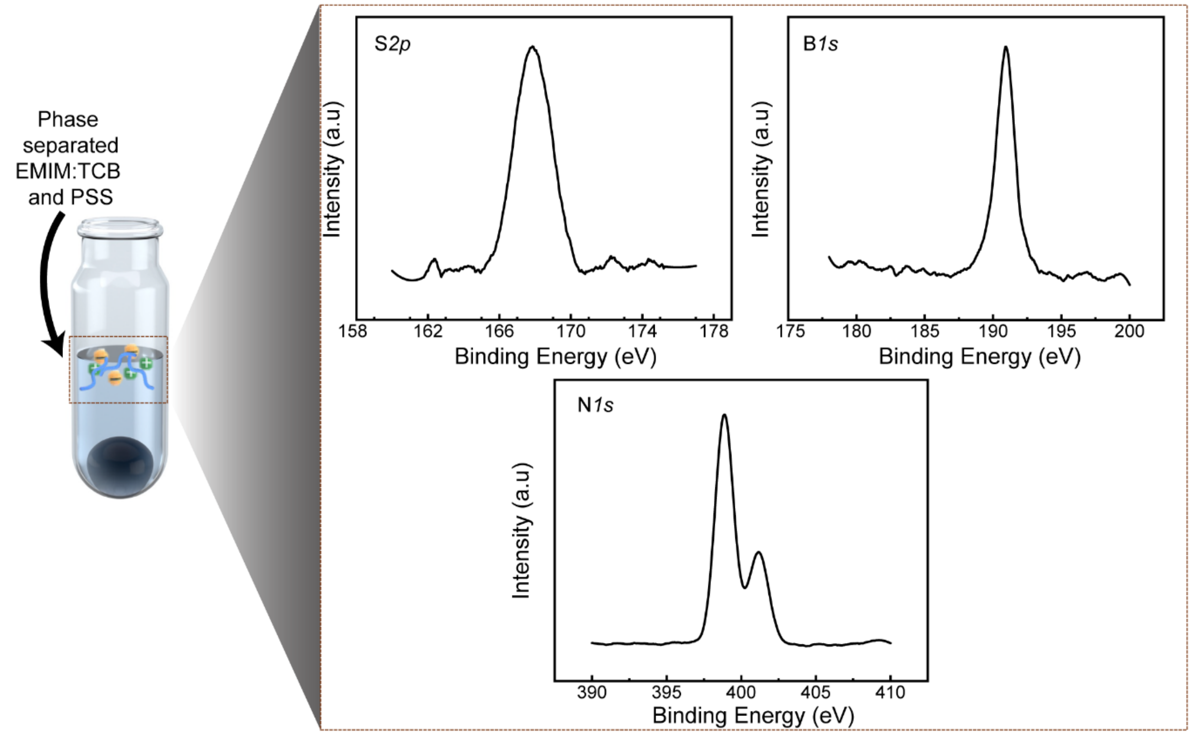

Supplementary Fig. 12 | XPS analysis of PILC after centrifugal purification. S *2p* (top left), B *1s* (top right) and N *1s* (bottom middle) peaks reflect the phase separation of water-soluble PSS and ionic liquid in the supernatant.

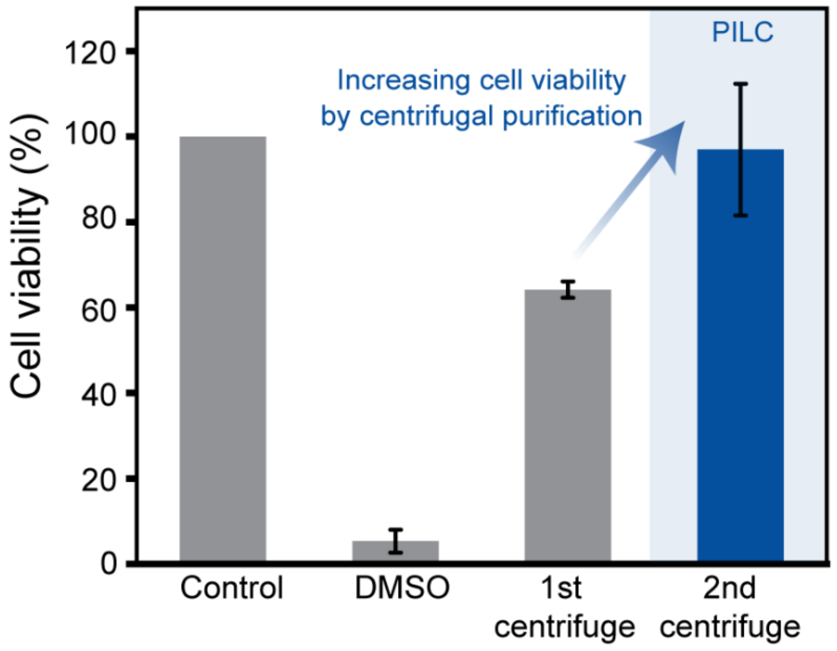

Supplementary Fig. 13 | Biocompatibility of the PILC ink after centrifugal purification. Cell viability of the PILC ink after centrifuging one and two times. DMSO is plotted as a negative control group. Data represents as mean values ± standard deviation (n = 4 independent material samples for control, DMSO and 1st centrifuge groups, n = 3 for 2nd centrifuge group).

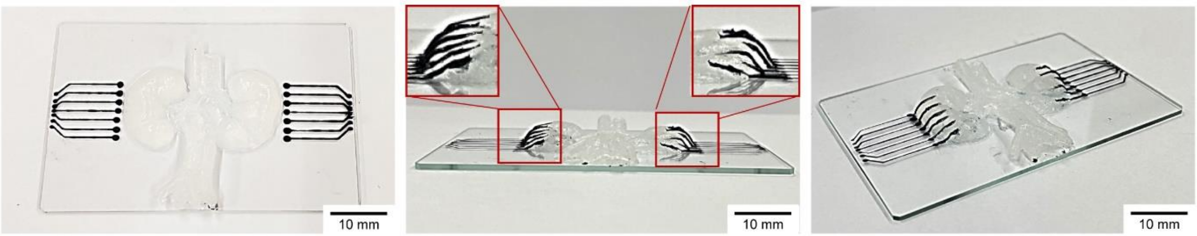

Supplementary Fig. 14 | 3D diagonally printed circuits lines for achieving an artificial 3D tissue structure with artificial kidney made of Ecoflex-0020. Photo of two different interfaces between 2D PILC lines and an artificial kidney made of Ecoflex-0020 (left), Photo of 3D diagonally suspended circuit lines printed onto the artificial kidney (middle and right) with diagonal overhang : ~ 6 mm with various printing angle.

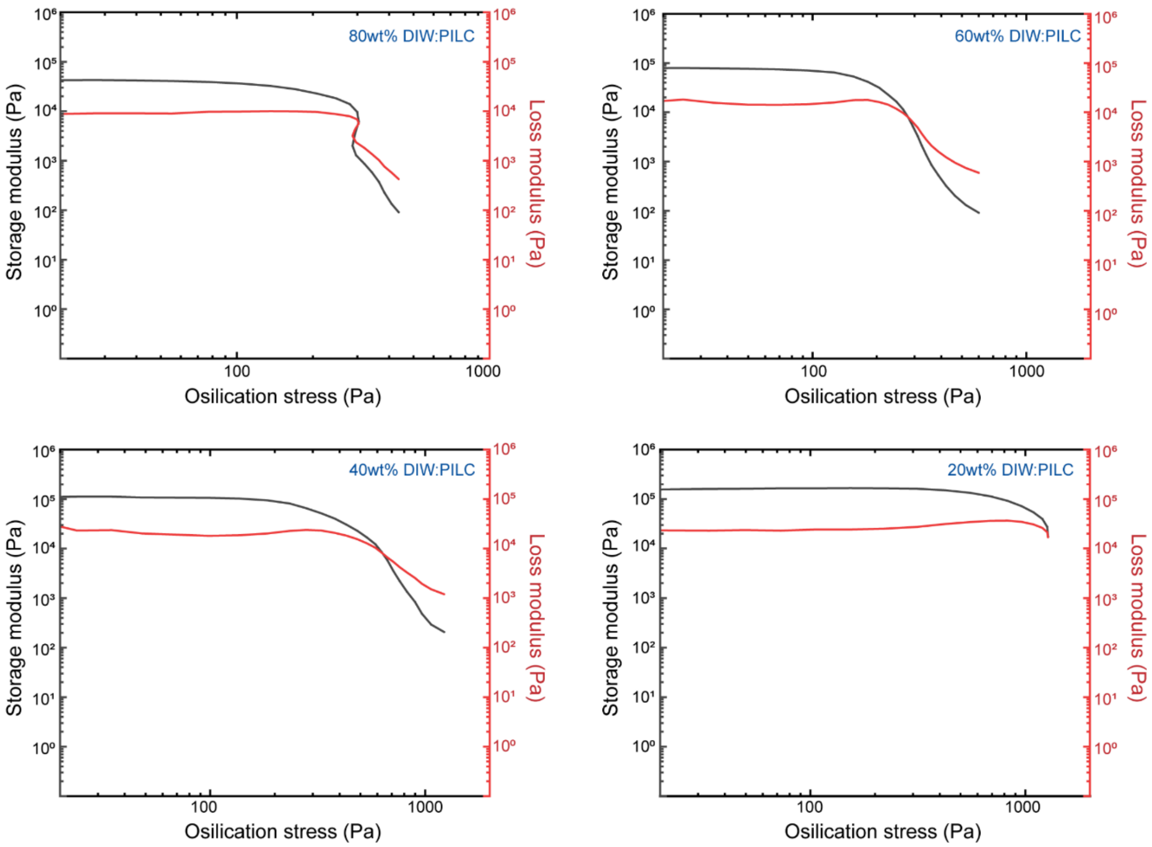

Supplementary Fig. 15 | Rheological characterization of the PILC inks with varying concentrations. The storage and loss modulus plots as a function of shear yield stress for different dilution ratio of 80 wt% (top left), 60 wt% (top right), 40 wt% (bottom left) and 20 wt% (bottom right) with PILC inks.

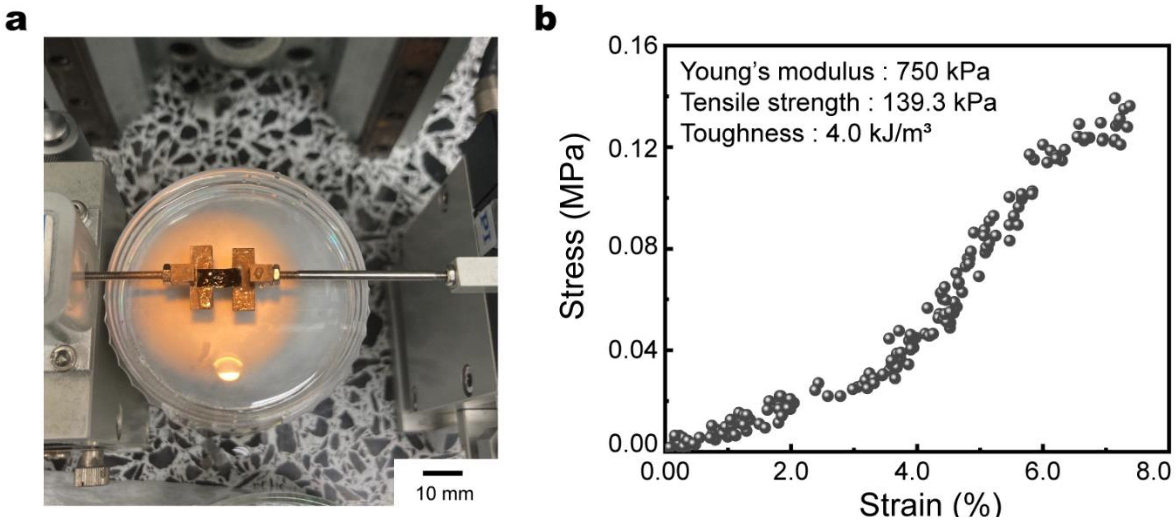

Supplementary Fig. 16 | Mechanical properties of freestanding PILC films. a, A photograph of the set-up used to measure the mechanical properties of the PILC ink on water. **b,** Stress versus strain of the PILC film. Printed PILC films demonstrate low Young's modulus (750 kPa) similar to tissues.

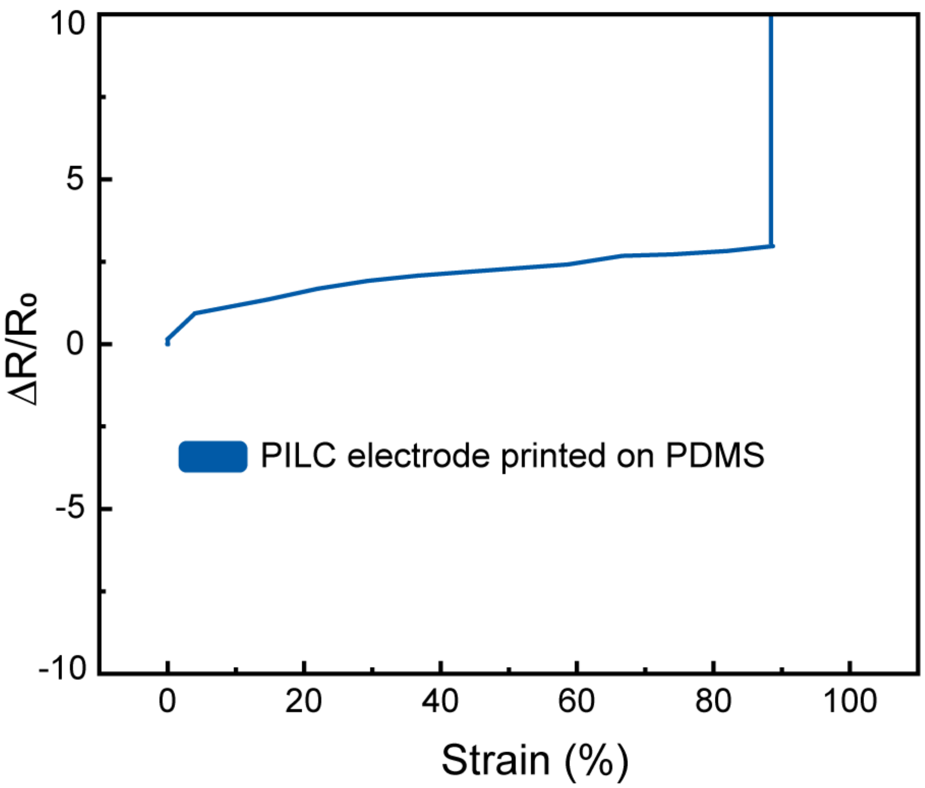

Supplementary Fig. 17 | Mechanical properties of printed PILC on a PDMS substrate at strain rate: 50 mm/min. Change in resistance as a function of strain for PILC electrode printed on PDMS.

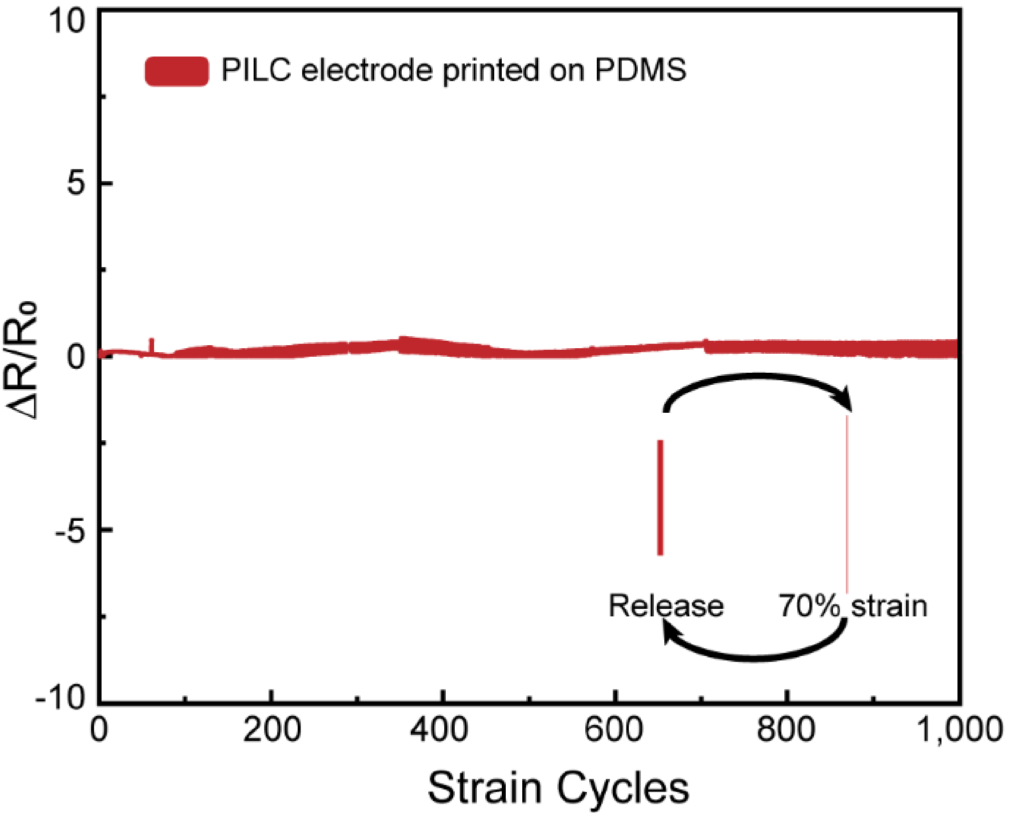

Supplementary Fig. 18 | Repeatability of printed PILC on a PDMS substrate for 1,000 cycles @ 70% strain at strain rate: 50 mm/min). Change in resistance under repetitive strain cycle of PILC electrode printed on PDMS.

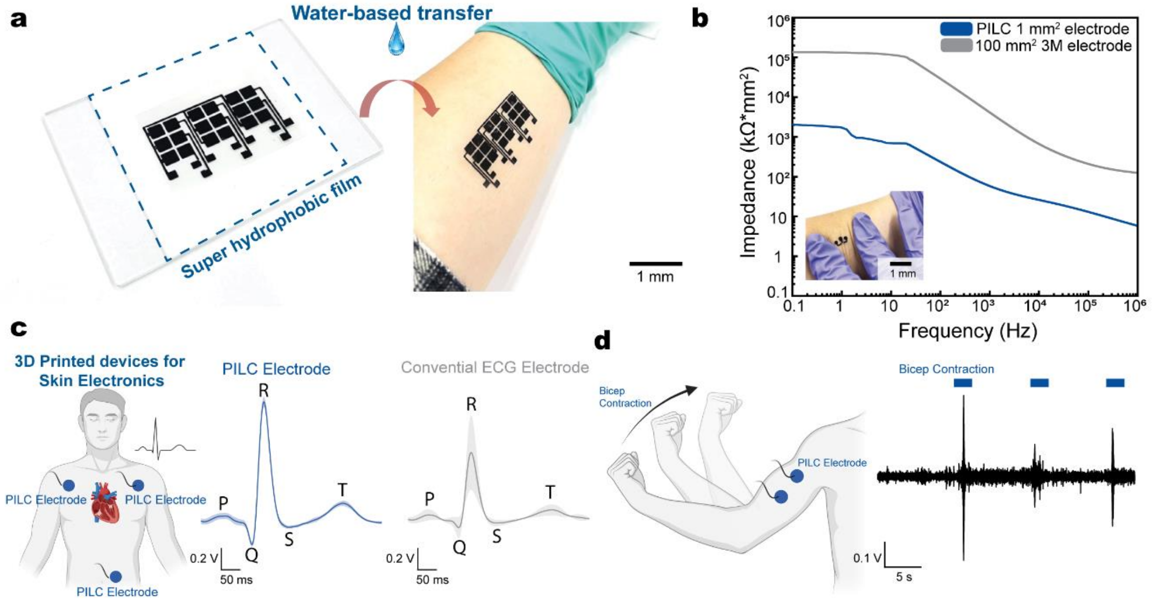

Supplementary Fig. 19 | 3D printed PILC electrodes for on-skin bioelectronics. **a**, Images of multielectrode EMG array printed with the PILC ink (left) and subsequent transfer to wet skin (right). **b**, Impedance spectra of PILC EMG electrodes in comparison to commercial 3M electrodes. **c**, Schematic (left) and average ECG waveform of the PILC ECG electrodes (middle) and conventional 3M electrodes (right). **d**, Schematic (left) and EMG waveform (right) collected with PILC EMG electrodes during bicep contraction. Supplementary Figure 19/panels c and d Created with BioRender.com released under a Creative Commons Attribution-NonCommercial-NoDerivs 4.0 International license.

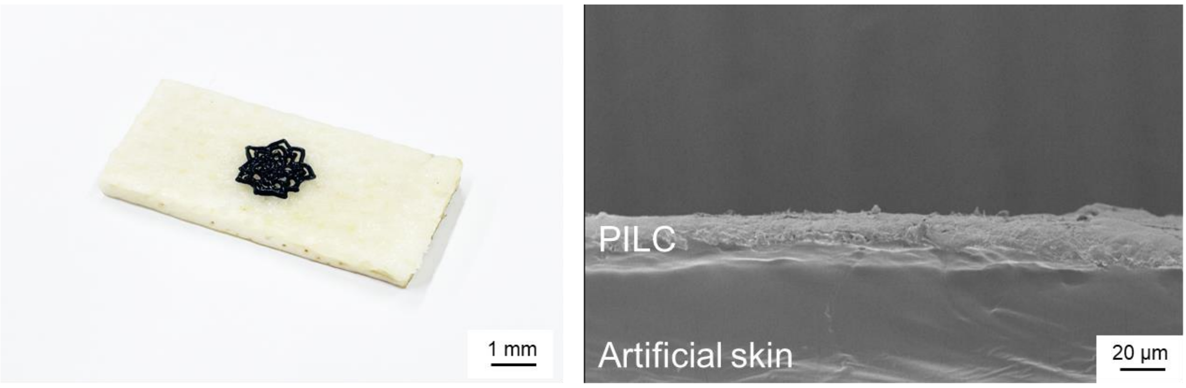

Supplementary Fig. 20 | Transfer of the printed PILC electrodes onto wet porcine skin. An image of the printed PILC electrodes transferred onto wet porcine skin (left) and a SEM image of transferred PILC onto artificial skin (right, Polyacrylamide hydrogel).

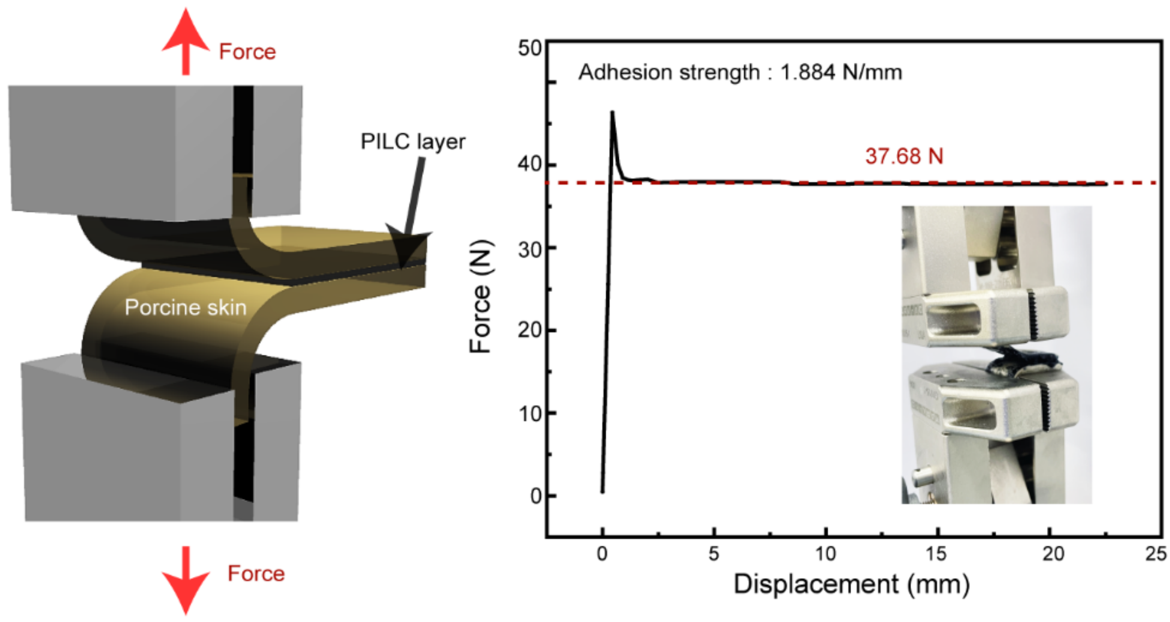

Supplementary Fig. 21 | Adhesion property of PILC between wet porcine skins. Schematic illustration of the method for measuring the adhesion property of PILC (left) and a plot representing the adhesion force of PILC versus displacement between porcine skin (right).

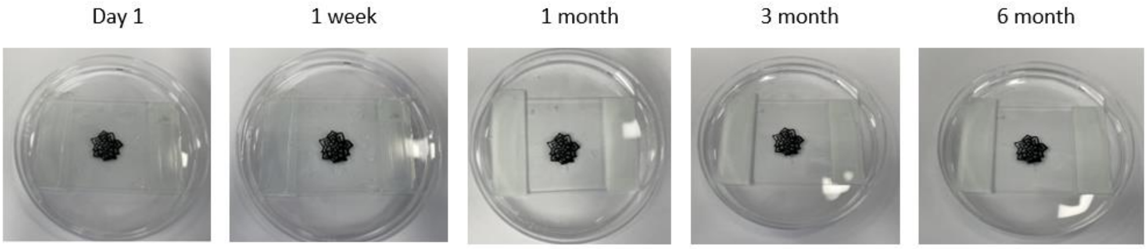

Supplementary Fig. 22 | Chemical stability of the printed PILC electrodes. Image of the printed PILC electrodes submerged in wet environment for 6 months (7.4 pH Phosphate Buffered Saline (PBS) solution at 37° C).

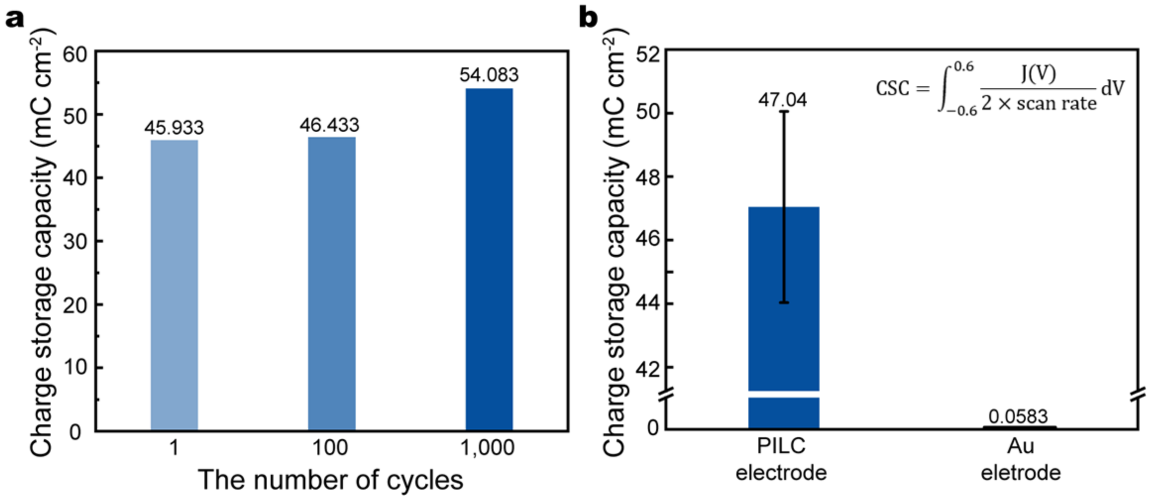

Supplementary Fig. 23 | Charge storage capacity (CSC) for printed PILC electrodes in comparison to conventional metal electrodes. a, CSC of printed PILC electrodes over 1,000 cycles. **b,** A plot to compare CSC between printed PILC electrodes and gold electrodes. The PILC electrode demonstrates increased CSC (47.04 mC/cm^2) in comparison to the gold electrode (0.0583mC/cm^2). Data represents as mean values ± SD (n = 3 independent experiments).

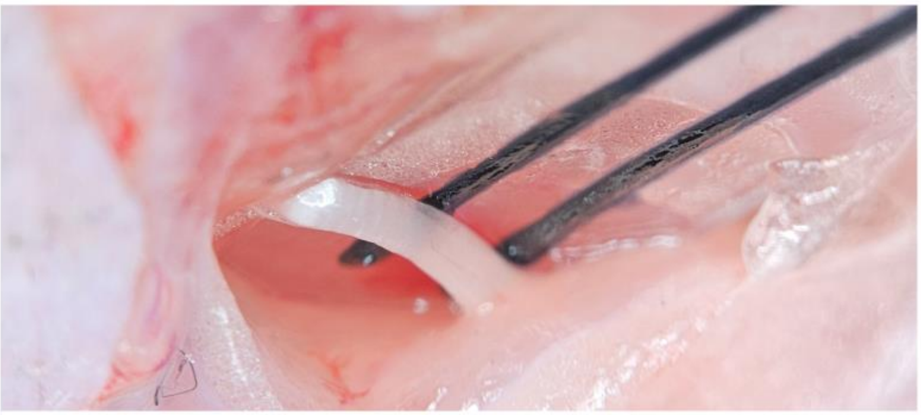

Supplementary Fig. 24 | Image of the PILC electrode utilize to interface with the sciatic nerve in an in vivo mouse model. Data reproducibility was confirmed by three independent experiments.

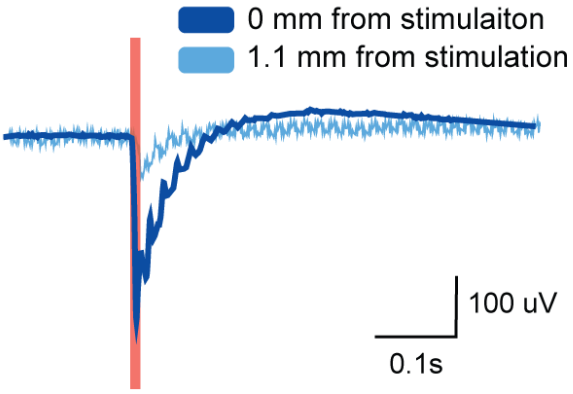

Supplementary Fig. 25 | Spatial selectivity of ECoG optically evoked potentials in Thy1-Chr2 transgenic mice. Optically-evoked EcoG waveforms collected with PILC ECoG device at the different site of optical stimulation.

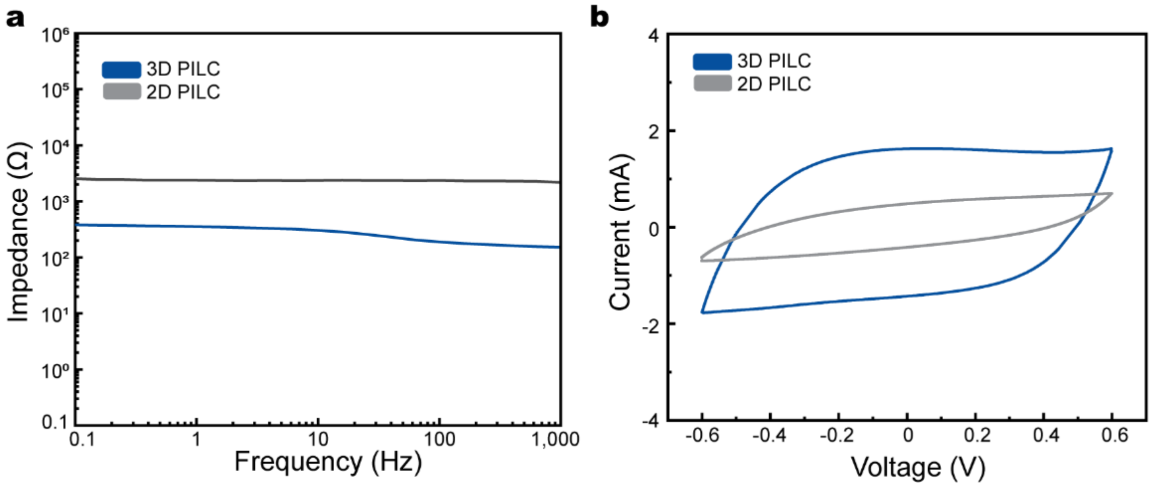

Supplementary Fig. 26 | Impedance and Charge storage capacity (CSC) for 3D-printed and 2D printed PILC electrode. a, A plot to compare Impedance between 3D-printed and 2D-printed PILC electrodes. **b,** A plot to compare CSC between 3D-printed and 2D-printed PILC electrodes. The 3D printed PILC electrode demonstrates increased impedance (151 Ω at 1kHz) and CSC (104.827 mC/cm^2) in comparison to 2D printed PILC electrodes (>1k Ω at 1kHz and 47.04 mC/cm^2).

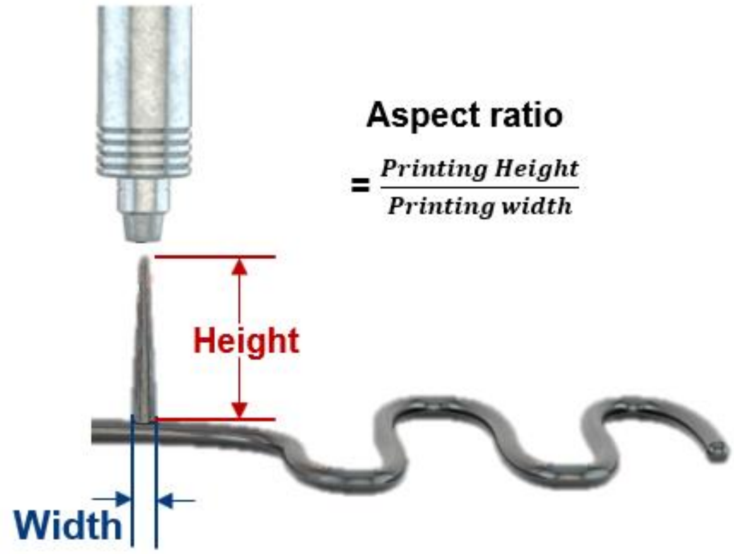

Supplementary Fig. 27 | Calculation method of 3D Aspect ratio. Schematic illustration of the method for calculating the aspect ratio.

Reference

1. Yuk, H. et al. 3D printing of conducting polymers. *Nat Commun* **11**, 1604 (2020).

2. Xie, X. et al. Liquid-in-liquid printing of 3D and mechanically tunable conductive hydrogels. *Nat Commun* **14**, 4289 (2023).

3.Ghaderi, S., Hosseini, H., Arash Haddadi, S., Kamkar, M. & Arjmand, M. 3D printing of solvent-treated PEDOT:PSS inks for electromagnetic interference shielding. *J. Mater. Chem. A*. **11**, 16027–16038 (2023).

4. Xing, W. et al. Omnidirectional Printing of PEDOT:PSS for High-Conductivity Spanning Structures. *ACS Appl. Mater. Interfaces*. **15**, 57717-57725 (2023)

5.Zhou, T. et al. 3D printable high-performance conducting polymer hydrogel for all-hydrogel bioelectronic interfaces. *Nat. Mater*. **22**, 895–902 (2023).

6. Yu, J. et al. 3D Printing of Robust High-Performance Conducting Polymer Hydrogel-Based Electrical Bioadhesive Interface for Soft Bioelectronics. *Small*, 2308778.

7. Hill, I. M. et al. Imparting High Conductivity to 3D Printed PEDOT:PSS. *ACS Appl. Polym. Mater*. **5**, 3989–3998 (2023).

8. Wang, F. et al. 3D Printed Implantable Hydrogel Bioelectronics for Electrophysiological Monitoring and Electrical Modulation. *Adv. Funct. Mater.* 2314471 (2023).

9. Lu, B. et al. Pure PEDOT:PSS hydrogels. *Nat. Commun*. **10**, 1043 (2019).

10. Chong, J. *et al.* Highly conductive tissue-like hydrogel interface through template-directed assembly. *Nat. Commun* **14**, 2206 (2023).

11. Won, D. *et al.* Digital selective transformation and patterning of highly conductive hydrogel bioelectronics by laser-induced phase separation. *Sci. Adv.* **8**, eabo3209.

12. Wei, H. *et al.* Orthogonal photochemistry-assisted printing of 3D tough and stretchable conductive hydrogels. *Nat Commun* **12**, 2082 (2021).

13. Zhang, J. et al. Engineering Electrodes with Robust Conducting Hydrogel Coating for Neural Recording and Modulation. *Adv. Mater*. **35**, 2209324 (2023).

14. Yao, B. et al. Ultrastrong, highly conductive and capacitive hydrogel electrode for electron-ion transduction. *Matter* 5, 4407–4424 (2022).
