# Supplementary material for: 3D printable and biocompatible PEDOT:PSS-ionic liquid colloids with high conductivity for rapid on-demand fabrication of 3D bioelectronics
Source: Nat Commun. 2024 Jul 11;15:5839. doi: 10.1038/s41467-024-50264-6 (PMC11239939; doi:10.1038/s41467-024-50264-6)
Supplement: Supplementary file 3 — Description of Additional Supplementary Information [file 41467_2024_50264_MOESM3_ESM.docx]

**Description of Additional Supplementary Files**

File Name: Supplementary Video 1

Description: High-resolution printing of PILC inks.

File Name: Supplementary Video 2

Description: Omnidirectional printing of PILC inks.
